# Biosynthesis and characterization of silver nanoparticles induced by fungal proteins and its application in different biological activities

**DOI:** 10.1186/s43141-019-0008-1

**Published:** 2019-11-01

**Authors:** Abdelmageed M. Othman, Maysa A. Elsayed, Naser G. Al-Balakocy, Mohamed M. Hassan, Ali M. Elshafei

**Affiliations:** 1 Microbial Chemistry Department, Genetic Engineering and Biotechnology Research Division, National Research Centre, 33 El Bohouth St., Dokki, Giza, 12622 Egypt; 2 Protein and Manmade Fibers Department, Textile Research Division, National Research Centre, 33 El Bohouth St., Dokki, Giza, 12622 Egypt

**Keywords:** Silver nanoparticles, *Aspergillus fumigatus*, Biosynthesis, Characterization, Antimicrobial, Antitumor

## Abstract

**Background:**

The present study aims to apply an efficient eco-friendly and inexpensive process for green synthesis of silver nanoparticles (AgNPs) through the mediation of fungal proteins from *Aspergillus fumigatus* DSM819, characterization, and its application as antimicrobial finishing agent in textile fabrics against some infectious microorganisms.

**Results:**

Optimum conditions for AgNP biosynthesis could be achieved by means of using 60% (v/v) of cell-free filtrate (CFF) and 1.5 mM of AgNO_3_ at pH 10.0 after 90 min. The obtained AgNPs were of spherical shape with 90% of distribution below than 84.4 nm. The biosynthesized AgNPs exerted an antimicrobial activity against the studied pathogenic microorganisms (*E. coli*, *B. mycoides*, and *C. albicans*). In addition, IC_50_ values against in vitro tumor cell lines were found to be 31.1, 45.4, 40.9, and 33.5 μg/ml for HCT116, A549, MCF7, and PC3, respectively. Even with a very low concentration (0.25%), the treated PET/C fabrics by AgNPs exerted an antimicrobial activity against *E. coli*, *B. mycoides*, and *C. albicans* to give inhibition zone diameter of 15, 15, and 16 mm, respectively.

**Conclusions:**

The green biosynthesis approach applied in this study is a non-toxic alternative to the traditional chemical and physical methods, and would be appropriate for biological large-scale production and prospective treatments.

**Graphical abstract:**

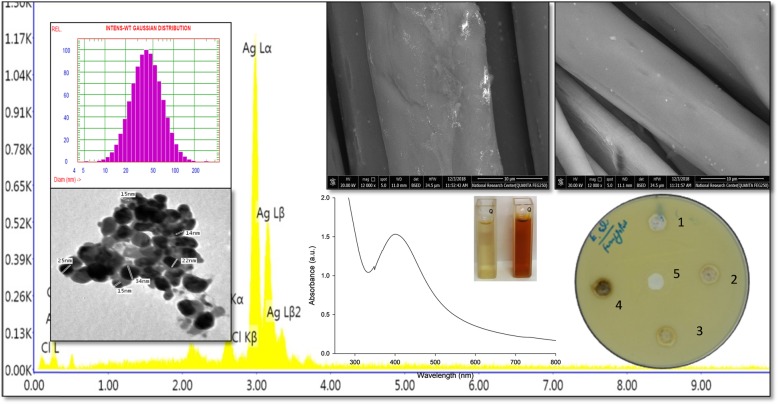

## Background

The advantages of using microbiological synthesis of silver nanoparticles (AgNPs) over chemical method has gained more importance during the last few years due to its higher and faster production and being lower cost and eco-friendly [33]. Generally, the synthesis of nanoparticles (NPs) received particular attention by the scientific community due to their unique properties and for their technological applications which reflect positively in improving many sectors of economy including pharmaceutics, cosmetics, industry, energy, and agriculture [22].

Metal nanoparticles (NPs) are very interesting to scientists as they bridge the gap between the bulk and atomic structures due to their physicochemical properties such as high surface area, precise pore size, and high reactivity [31]. Among the noble metal, silver plays a major role in medicine as it has been proven that silver was used for preventing bacterial infections and also exhibits wound healing activity. Investigations on silver nanoparticles (AgNPs) and their colloidal form exhibit catalytic and antibacterial properties, high conductivity, and chemical stability [29].

AgNPs can be prepared by various methods such as biological, physical, chemical, and other electrochemical, photochemical, sonolytic, and radiolytic methods [17]. Biological methods were found to be the most proper technique for the preparation of AgNPs, as NPs produced have a longer shelf life and stability as natural capping takes place. The green biosynthesis of NPs has a longer shelf life, cost effective and simple downstream processing, and effective purification methods. Plant extracts, bacteria, and fungi are the main sources involved in the biosynthesis of AgNPs [3].

Other sources for AgNP synthesis using biological materials such as honey [27], milk [12], coconut water [8], and egg white [14] were reported. Otunola and Afolayan [25] reported the synthesis, characterization, and biological properties of silver nanoparticles (AgNPs) using the extract from garlic, ginger and cayenne pepper (1:1:1; w/w/w), and silver nitrate solution. Different fungi were able to effectively synthesize AgNPs. Mukherjee et al. [20] reported that *Trichoderma asperellum* synthesizes silver nanoparticles in the range of 13–18 nm when subjected to AgNO_3_ solution. Patil [26], Mukherjee et al. [19], and Kathiresan et al. [10] reported the formation of AgNPs using *Fusarium semitectum*, *Verticillium* sp., and *Penicillium fellutanum*, respectively. An efficient eco-friendly synthesis of silver nanoparticles (AgNPs) using aqueous culture filtrate of *Pestalotiopsis microspora* has been reported. The analysis of UV–visible confirmed AgNP formation at absorption peak at 435 nm [21].

In the present work, silver nanoparticles (AgNPs) synthesized by a microbiological method using *Aspergillus fumigatus* were applied in textile fabrics and tested as antimicrobial finishing agent against some infectious microorganisms as well as an antitumor agent using different tumor cell lines.

## Methods

### Chemicals

Nutrient agar medium (70148 nutrient agar) was provided by Fluka, Spain. Glucose was purchased from Koch-Light Lab., England. Silver nitrate was provided by Sisco Research lab, India. Potassium chloride was obtained from Merck, Germany. Agar–Agar was obtained from Fluka, France. Polyester/cotton blend (PET/C 50/50) fabrics in form of filament woven fabric cloth made from filament yarns was kindly supplied by Misr Polyester Co., Kafr EL-Dwar, Egypt. Acid cellulase enzyme (Cellusoft® L) used for fabric pretreatment was obtained from Novo Nordisk, Denmark.

### Microorganisms

*Aspergillus fumigatus* DSM819 was cultured and stored on modified Czapek–Dox’s solid medium slants and refreshed before use. For the purpose of antimicrobial determinations, *Escherichia coli*, *Bacillus mycoides*, and *Candida albicans* were applied as representative microorganisms of Gram-negative bacteria, Gram-positive bacteria, and non-filamentous fungi. They were maintained on nutrient agar medium with the following composition (g/l): peptone, 3; yeast extract, 1.5; meat extract, 1.5; glucose, 0.5; NaCl, 0.25; and agar, 20.0 at pH 7.0. All microbial cultures applied in this study were obtained from the culture collection of Microbial Chemistry Dept., NRC, Egypt.

### Preparation of cell-free filtrate and AgNP biosynthesis

*A. fumigatus* DSM819 was cultivated in 250 ml Erlenmeyer conical flasks which contained 50 ml of the modified Czapek–Dox’s liquid medium (g/l): NaNO_3_, 2; KH_2_PO_4_, 1; MgSO_4._7H_2_O, 0.5; KCl, 0.5; and glucose, 20 for 6 days at 28 °C under static conditions. After that, the culture was filtered via Whatman No. 1 filter paper and the resulted supernatant (cell-free filtrate; CFF) was applied for the mediation of AgNP biosynthesis. The obtained CFF was applied in various reaction mixtures containing silver nitrate aqueous solution (1 mM) as silver source for AgNP biosynthesis process. Then, the mixtures were incubated in the dark (to avoid the photoactivation of silver nitrate) at 30 °C and continues shake at 100 rpm. After incubation, the absorbance of the characteristic reddish brown color of the biosynthesized AgNPs was scanned using UV–visible spectrophotometer (Cary 100 UV-Vis; Agilent Technologies, Germany). Cell-free filtrates as well as silver nitrate solution (1 mM) were used as controls.

### Optimization of AgNP biosynthesis

The CFF obtained from *A. fumigatus* DSM819 culturing was added at different concentrations in ratios between 10 and 60% (v/v), which means adding CFF volumes between 0.5 and 3.0 ml to a total reaction mixture of 5.0 ml, while keeping the AgNO_3_ concentration at a level of 1.0 mM. The pH value effect was studied through preparing different reaction mixtures adjusted at pH values ranging between 9.0 and 11. The effect of reaction time on the AgNP biosynthesis process was evaluated by incubating the reaction mixtures at optimum conditions for 10, 20, 30, 45, 60, 90, and 120 min. The effect of silver ions on the biosynthesis of AgNPs by *A. fumigatus* DSM819 was determined by varying the AgNO_3_ concentration to range from 0.5 to 1.5 mM. All experiments were carried out in triplicates, and the average data was presented.

### Characterization of the biosynthesized AgNPs

The UV–visible spectra of AgNPs were recorded as a function of wavelength using UV/vis spectrophotometer (Cary 100 UV-Vis; Agilent Technologies, Germany) operated at data interval of 1.0 nm. The AgNP solution was centrifuged for 20 min at 10000 rpm and was drop coated on a carbon-coated copper grid and dried to be applied for studying both the scanning electron microscopic (SEM) and elemental analysis of the biosynthesized AgNPs using scanning electron microscope (SEM-Quanta FEG250) operated at an accelerating voltage of 20 kV and coupled with energy dispersive X-ray analysis (EDAX) for compositional analysis and the conformation of presence of elemental silver.

The shape and size of AgNPs were determined by transmitting electron microscope (TEM). For TEM, a drop of aqueous AgNP sample was loaded on a carbon-coated copper grid, and it was allowed to dry at room temperature; the micrographs were obtained using TEM (JEOL JEM-1230) at 160 kV. Particle size was measured on a dynamic light scattering (DLS) instrument (PSS, Santa Barbara, CA, USA), using the 632-nm line of a HeNe laser as the incident light with angel 90°.

For Fourier transform infrared (FTIR) spectroscopy measurements, reaction mixtures containing AgNO_3_ at a concentration of 1.5 mM and *A. fumigatus* DSM819 CFF were prepared and incubated for 90 min at pH 10 to form AgNPs, then centrifuged at 10,000 rpm for 15 min and re-dispersed in sterile distilled water. The process of centrifugation and re-dispersion was repeated four times to ensure good separation of the AgNPs from other contaminants. The obtained pellets were then dried, and the powders were subjected to FTIR spectroscopy measurement. These measurements were carried out on a JASCO FTIR (Japan) instrument in the diffuse reflectance mode at a resolution of 4 cm^−1^ in KBr pellets.

### Cytotoxic effect on human cell lines

Cell viability was assessed by the mitochondrial-dependent reduction of yellow MTT (3-(4,5-dimethylthiazol-2-yl)-2,5-diphenyl tetrazolium bromide) to purple formazan [18] in a sterile area using a Laminar flow cabinet biosafety class II level (Baker, SG403INT, Sanford, ME, USA). Cells (as a gift from Dr. Stig Linder, Karolinska institutet, Solna, Sweden) were suspended in RPMI 1640 medium, 1% antibiotic–antimycotic mixture (10,000 U/ml potassium penicillin, 10,000 μg/ml streptomycin sulfate, and 25 μg/ml amphotericin B), and 1% l-glutamine at 37 °C under 5% CO_2_. Cells were batch cultured for 10 days, then seeded at concentration of 10 × 10^3^ cells/well in fresh complete growth medium in 96-well microtiter plastic plates at 37 °C for 24 h under 5% CO_2_ using a water-jacketed carbon dioxide incubator (Sheldon, TC2323, Cornelius, OR, USA). Media was aspirated, fresh medium (without serum) was added, and cells were incubated either alone (negative control) or with different concentrations of sample to give a final concentration (54.0, 27.0, 13.5, 6.75, 3.37, 1.68, 0.84, and 0.40 μg/ml). After 48 h of incubation, medium was aspirated, and 40 μl MTT salt (2.5 μg/ml) was added to each well and incubated for further 4 h at 37 °C under 5% CO_2_. To stop the reaction and dissolving the formed crystals, 200 μl of 10% sodium dodecyl sulfate (SDS) in deionized water was added to each well and incubated overnight at 37 °C. A positive control was used as a known cytotoxic natural agent who gives 100% lethality under the same conditions. The absorbance was measured using a microplate multi-well reader (Bio-Rad Laboratories Inc., model 3350, Hercules, CA, USA) at 595 nm and a reference wavelength of 620 nm. A statistical significance was tested between samples and negative control (cells with vehicle) using independent *t* test by SPSS 11 program. DMSO is the vehicle used for dissolution with final concentration on the cells which was less than 0.2%. The percentage of change in viability was calculated according to the formula: ((Reading of extract/Reading of negative control) − 1) × 100. A probit analysis was carried for IC_50_ and IC_90_ determination using SPSS 11 program.

### Pretreatment of PET/C fabrics by cellulase

Using a high-temperature high-pressure laboratory dyeing machine, cellulase (3%) was placed in stainless-steel bowls, then the fabrics were immersed in the solutions (pH 4.5), and the sealed bowls were rotated in a closed bath containing ethylene glycol at 45 °C. The material to liquor ratio was 1:15. The bath temperature increased at a rate of 5 °C/min, and after 40 min, the enzymatic treatment was then terminated by raising the pH to 10 by using Na_2_CO_3_. The fabric samples were removed from the bath, rinsed repeatedly with distilled hot and cold water, and then allowed to dry in the open air.

### PET/C fabric treatment by the biosynthesized AgNPs

The activated PET/C blend fabric by cellulase and un-hydrolyzed fabrics (blank without enzymatic treatment) were immersed in the biosynthesized AgNP dispersion, and then, the samples were squeezed to a pickup of 60% (wt/wt) of the solution, dried in air at 22 °C for 24 h, and finally cured in an oven at 140 °C for 10 min. In order to evaluate the AgNP adhesion to the PET/C blend fabrics, the treated fabrics were washed five times according to a standard method AATCC Test Method (61-1989). The chemical structure was determined using FTIR spectrometer (model NEXUS 670, NICOLET, USA) in spectral range from 4000 to 400 cm^−1^.

### Assessment of antimicrobial activity

For the assessment of AgNP antimicrobial activity, agar diffusion method was applied. The microbial strains were grown (seeding inoculation technique using 100 μl of re-suspended overnight culture at 37 °C (1 × 10^7^ CFU/100 μl)) at 37 °C on nutrient agar medium (70148 nutrient agar, Fluka, Spain) with the subsequent composition (g/l): peptone, 5.0; NaCl, 5.0; yeast extract, 2.0; meat extract, 1.0; and agar 15.0 (pH 7.0). AgNP samples (108 μg/ml) obtained from the reaction of *A. fumigatus* DSM819 CFF and AgNO_3_ at different pH values were applied in a volume of 250 μl into the pre-made holes in Petri dishes of the previously mentioned nutrient agar medium, whereas in case of PET/C fabrics treated by the biosynthesized AgNPs, circular discs of 12 mm from the treated fabrics were applied. Culture plates were left overnight at 4 °C, and then, plates were incubated for 7 h at 37 °C. The developed inhibition zones were measured based on AATCC test method [7].

## Results

### Biosynthesized AgNP UV–visible spectroscopy

**Fig. 1:**
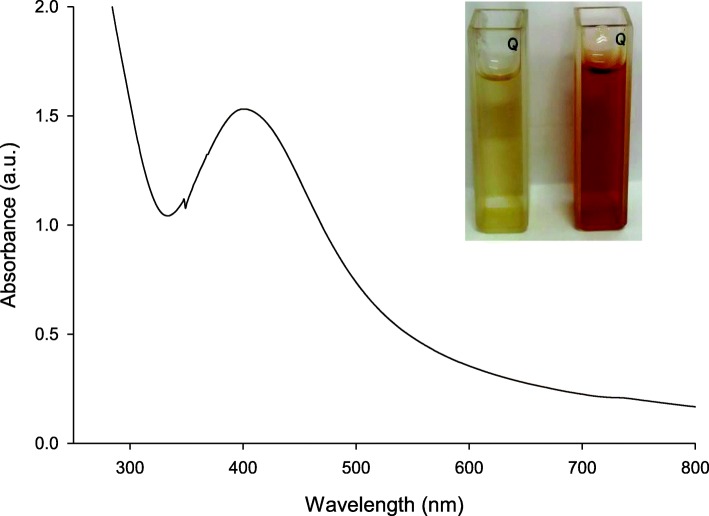
UV–visible absorption spectrum of the biosynthesized AgNPs. The inset photo shows the change of reaction mixture color to reddish brown as a result of AgNP formation

The UV–visible spectroscopy results using *A. fumigatus* DSM819 filtrate indicate the ability of *A. fumigatus* DSM819 to form AgNPs extracellulary through reduction of Ag^+^ to form the AgNPs. Figure 1 shows the UV–vis spectroscopy scan of AgNP formation, and this peak is due to the change of reaction mixture color to reddish brown as a result of AgNP formation and SPR excitation. The distinct and typical SPR band for AgNPs was obtained in the region of 410 nm as a result of AgNP formation, in comparison to controls using CFF or silver nitrate solution separately, where there is no reddish brown color development and subsequently no typical SPR band for AgNPs, demonstrating the absence of abiotic reduction of silver nitrate.

### Optimization of AgNP biosynthesis

#### Effect of CFF ratio on AgNP biosynthesis

**Fig. 2:**
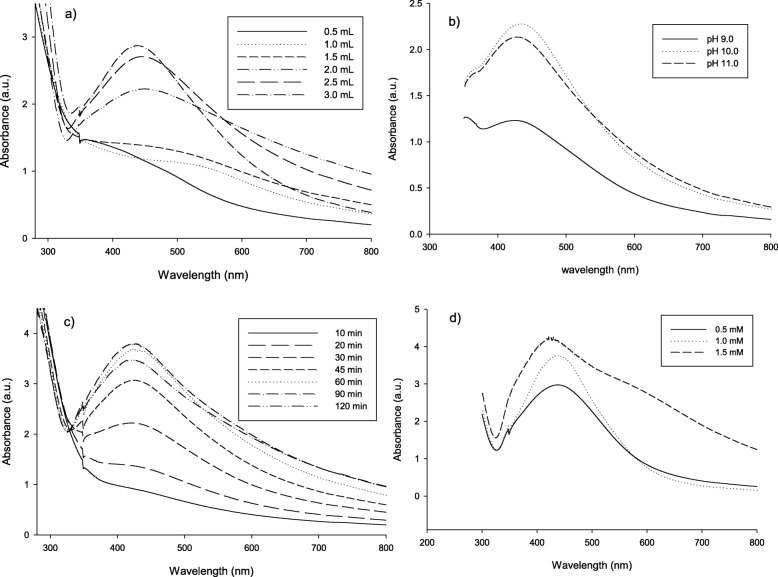
UV–visible absorption spectra of AgNP biosynthesis optimization at different: **a** CFF volumes, **b** pH values, **c** reaction time, and **d** silver nitrate concentrations

Different reaction mixtures in a total volume of 5.0 ml including silver nitrate (1 mM) and diverse ratios from CFF of *A. fumigatus* DSM819 ranging between 10 and 60% (v/v) were tested. Results obtained indicate that the formation of AgNPs is directly proportional to the ratio of *A. fumigatus* DSM819 CFF, with maximum ability to biosynthesize AgNPs at ratio of 60% (v/v). The ability of *A. fumigatus* DSM819 CFF to form AgNPs is gradually decreased by the decreasing of CFF ratio to give its lowest activity at a ratio of 10% (v/v) which were declared by the SPR scanning peaks that are illustrated in Fig. 2a.

#### Effect of pH value on AgNP formation

The results obtained demonstrate the influencing effect of pH value on the biosynthesis of AgNPs by mediation of *A. fumigatus* DSM819 CFF. The optimum pH value for AgNP formation (the highest SPR peak intensity) was recorded at pH 10.0 after investigating different reaction mixtures adjusted at different pH values (Fig. 2b). It is worthy to mention that the capability of the produced enzyme and protein as reducing and stabilizing agents appeared in alkaline medium, and when the reaction was performed at pH values lower than 9.0, no SPR peak was obtained. While, the adjustment of reaction mixture pH value to be higher than 11.0, an immediate precipitate of silver was obtained that impede the formation of SPR peak and hence the possibility of activity recording.

#### Effect of reaction time

The effect of reaction time on AgNP formation was investigated by incubating the reaction mixtures under optimum conditions for different time intervals ranging between 10 and 120 min. The obtained results indicated that the characteristic color intensity is directly proportional to the incubation time until 90 min which is the optimum time required for the maximum AgNP formation (Fig. 2c). After that, the increase in time course affected the formation process negatively as indicated in the peak of 120 min incubation time. Figure 2c also declares that the silver ion reduction velocity was slow for the first incubation durations (10–20 min) which were observed as small peaks at the region of activity (430 nm). After that, an increase in peak intensity was recorded after longer time periods up to 90 min due to the increase in numbers of AgNPs, and then, the peak intensity starts to go down at 120 min. Here, in the current case, 90 min is the optimum time for AgNP formation which is due to the exceptional reducing potential of the *A. fumigatus* DSM819 CFF active components.

#### Effect of silver nitrate concentration

The metal salt concentration has been revealed to influence nanoparticle biosynthesis effectively. The intensity of reaction color changed to reddish brown even at the lowest used concentration (0.5 mM) and become more dark reddish brown at higher concentrations of silver nitrate (1.0–1.5 mM). This developed color resulted to an increase in absorbance intensity up to 1.5 mM of AgNO_3_, which is the highest obtained peak strength (Fig. 2d). It is worthy to mention that by using higher silver nitrate concentrations over 1.5 mM, a sudden black precipitate from silver nanoparticles occurred which impedes the formation of activity peak, and hence, no typical SPR band for AgNPs was obtained in the region of 410 nm.

### Characterization of the biosynthesized AgNPs

#### SEM and EDAX profile of the biosynthesized AgNPs

**Fig. 3:**
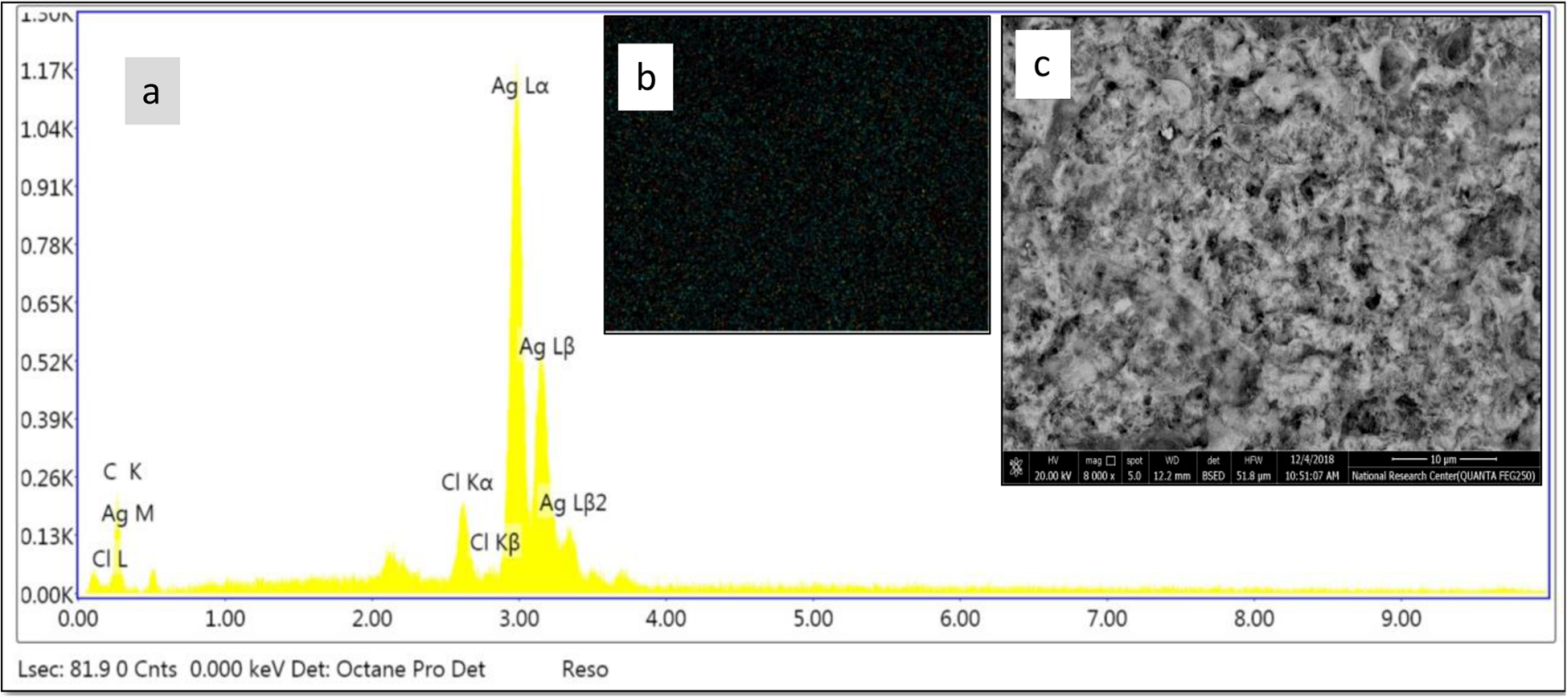
Profiles of the biosynthesized AgNPs by *A. fumigatus* DSM819 CFF. **a** EDAX profile of nanosilver and other combined metals. **b** Magnified SEM micrograph (× 8000). **c** EDAX mapping micrograph showing AgNP percentage as turquoise () dots in the total sample black background. Samples used were produced from reaction mixture containing 60% from *A. fumigatus* CFF and 1.5 mM AgNO_3_ and incubated for 90 min at pH 10

For the quantitative and qualitative evaluation of elements implicated in the process of nanoparticle formation, energy dispersive X-ray (EDAX) investigation usually applied. The profile obtained from SEM micrograph of the biosynthesized AgNPs by *A. fumigatus* DSM819 CFF at × 8000 magnification shows the homogeneity of the prepared sample (Fig. 3c). The resulted EDAX analysis established the incidence of elemental silver as strong signal energy peaks around 3.0 keV, verifying the AgNP biosynthesis successfully. In addition, (EDAX) profile of the biosynthesized sample indicates the presence of silver by 89.72, carbon 6.56, and chloride 3.72 (weight %), as well as EDAX mapping micrograph also confirming AgNP percentage in the biosynthesized sample as turquoise dots in the total sample black background (Fig. 3a, b).

#### TEM and DLS analyses

**Fig. 4:**
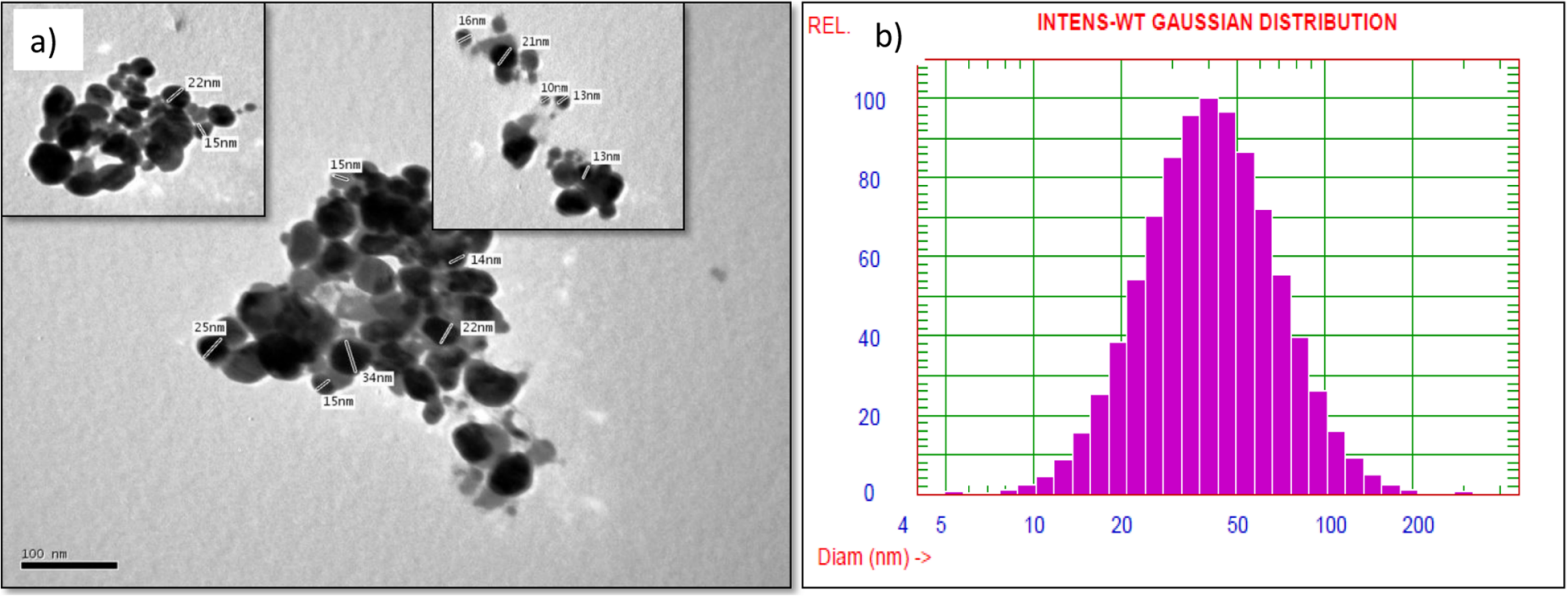
**a** TEM micrograph using JEOL (JEM-1230) Electron Microscope (150Kx). **b** Dynamic light scattering (DLS) of the biosynthesized AgNPs through the mediation by *A. fumigatus* DSM819 CFF

The obtained TEM profile reveals the spherical shape of the biosynthesized AgNPs as a result of treating silver nitrate solution by *A. fumigatus* DSM819 CFF. In addition, TEM profile shows the variation of the diameter range of the biosynthesized AgNPs between 10 and 34 nm (Fig. 4a). The obtained pictures show some individual silver particles in addition to some aggregates with dissimilar size ranges in indirect contact to ensure the nanoparticle stabilization by a capping agent. In order to get more precise view of particle size distribution, DLS technique was applied (Fig. 4b). Results obtained from DLS graph show that the biosynthesized AgNPs are in the nanoscale form with mean diameter of 46.2 nm and percentage of distribution as follows: 25% of distribution below than 30.6 nm, 50% of distribution below than 46.2 nm, 75% of distribution below than 62.2 nm, and 90% of distribution below than 84.4 nm.

#### FTIR spectroscopy

**Fig. 5:**
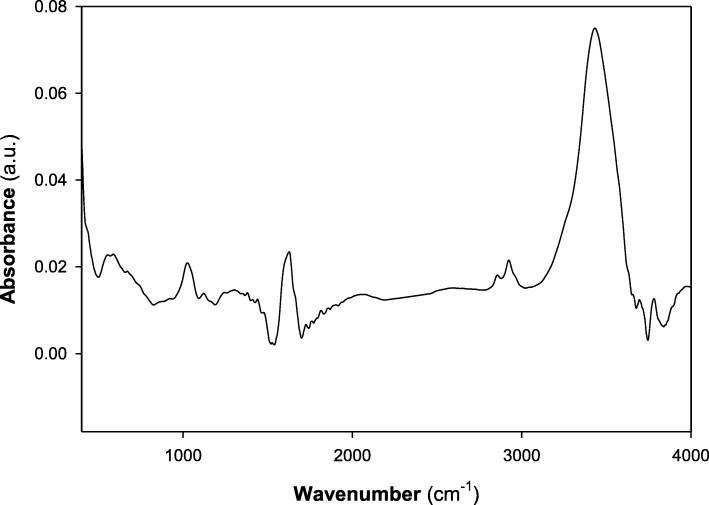
FTIR spectroscopic analysis of the biosynthesized AgNPs through the mediation by *A. fumigatus* DSM819 CFF

In order to recognize the chief functional groups accountable for the formation of AgNPs by *A. fumigatus* DSM819 CFF and have a role in its capping and stabilization, FTIR spectroscopic analysis was conducted. The obtained FTIR spectrum is illustrated in Fig. 5, which declares the appearance of several absorption peaks. In this regard, the appearance of the sharp peak at 3431 cm^−1^ is thought to be overlapping –NH stretching vibration which is a typical phenomenon for proteins. In addition, O–H stretching in flavonoids, alcohols, and phenolic compounds could be detected, and C–H stretching vibrations of methylene, methyl, and methoxy groups could be observed at 2923 and 2856 cm^−1^. The N–C=O amide bond of proteins due to carbonyl stretch in proteins was recorded at 1626 cm^−1^, while absorption peaks at 1121 and 1024 cm^−1^ can be due to stretching vibration of C–N aromatic and aliphatic amines.

### Antimicrobial activity

**Fig. 6:**
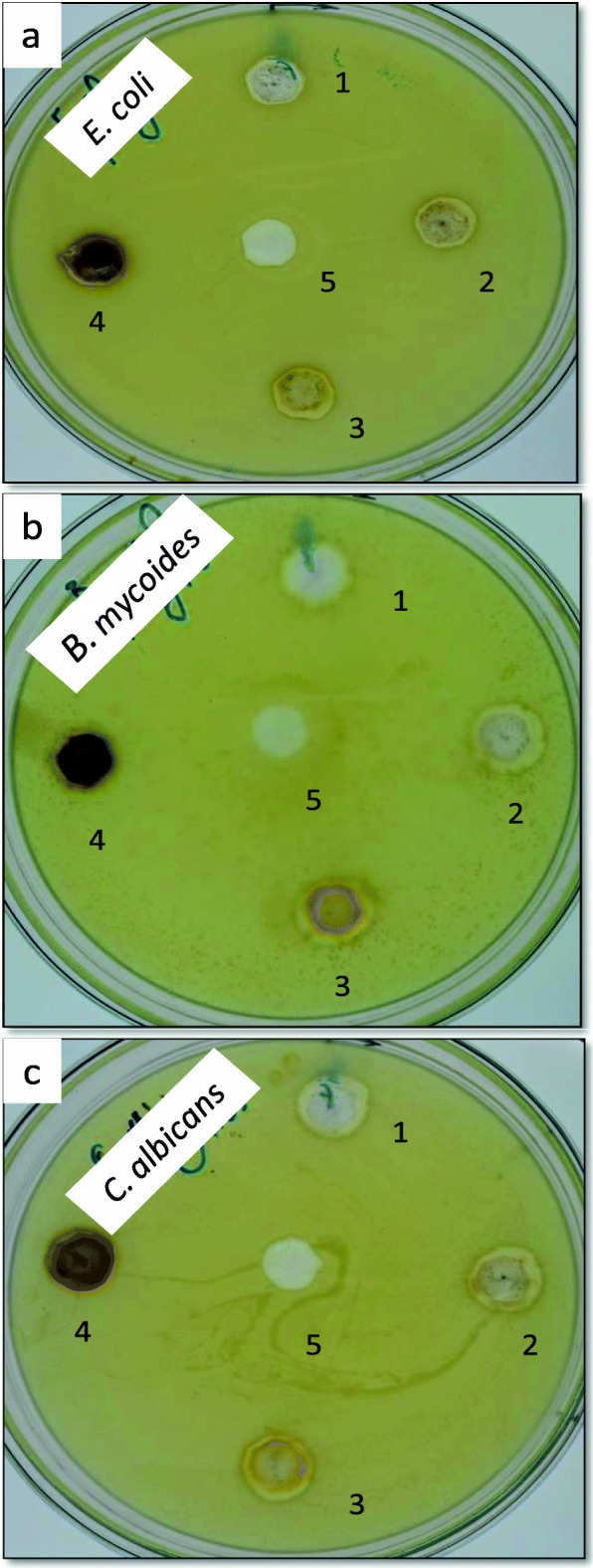
Antimicrobial activity of the AgNPs biosynthesized by means of *A. fumigatus* CFF mediation. (1) AgNPs sample formed at pH 6.0, (2) at pH 8.0, (3) at pH 10.0, (4) at pH 12.0, and (5) cell free filtrate

The biosynthesized AgNPs through the mediation by *A. fumigatus* DSM819 CFF at different pH values exerted an antimicrobial activity against the studied representative pathogenic microorganisms (*E. coli*, *B. mycoides*, and *C. albicans*) which depends on the microbe type and its cellular constituents. The exerted antimicrobial effect was measured as inhibition zone diameters (mm), where the effect against Gram-negative bacteria (*E. coli*) was the lowest one (15–16 mm) in comparison with Gram-positive bacteria (*B. mycoides*) which may owe to the dissimilarity in composition of the cell wall. In this regard, CFF as a control did not demonstrate any antimicrobial activity (Table 1 and Fig. 6). The antimicrobial activity in opposition to *Candida albicans* as pathogenic yeast was considerable and shows inhibition zone within the range against Gram-negative bacteria (Table 1 and Fig. 6).

**Table 1:** Antimicrobial activity of the AgNPs biosynthesized by means of *A. fumigatus* CFF mediation

Sample	Diameter of inhibition zones (mm)		
	*E. coli*	*B. mycoides*	*C. albicans*
Cell-free filtrate	0	0	0
pH 6.0	16	18	17
pH 8.0	16	18	18
pH 10	16	19	18
pH 12	15	16	15

### Antitumor activity of AgNPs

In vitro cytotoxicity of the biosynthesized AgNPs was evaluated against BJ1 (normal skin fibroblast), and its antitumor activity was assessed against HCT116 (human colon carcinoma), A549 (lung carcinoma cell line), MCF7 (human Caucasian breast adenocarcinoma), and PC3 (prostate cell line) at different concentrations (0.4 to 54 μg/ml) using MTT assay (Table 2). The results revealed that there was a straight dose–response association with tested cells at tested concentrations. In relation to cell fatality, lethal concentration of the biosynthesized AgNPs which causes the death of 50% of cells in 48 h (IC_50_) was found to be 31.1, 45.4, 40.9, and 33.5 μg/ml for HCT116, A549, MCF7, and PC3, respectively. In addition, IC_90_ was recorded to be 52.3, 72.8, 65.7, and 60.7 μg/ml for the previously mentioned cell lines, respectively. The biosynthesized AgNPs were able to restrain the cell line growth at low concentrations, where at a concentration of 54 μg/ml, they were able to inhibit 84.6, 60.8, 68.9, and 69.5% of HCT116, A549, MCF7, and PC3, respectively.

**Table 2:** Antitumor activity of the biosynthesized AgNPs

Cell line	Antitumor activity		
	IC_50_ (μg/ml)	IC_90_ (μg/ml)	Inhibition (%) at 54 ppm
BJ1 (normal skin fibroblast)	–	–	15.6
HCT116 (colon cell line)	31.1	52.3	84.6
A549 (lung carcinoma cell line)	45.4	72.8	60.8
MCF7 (human Caucasian breast adenocarcinoma)	40.9	65.7	68.9
PC3 (prostate cell line)	33.5	60.7	69.5

*IC*_*50*_ lethal concentration of the sample which causes the death of 50% of cells in 48 h, *IC*_*90*_ lethal concentration of the sample which causes the death of 90% of cells in 48 h

### Characterization of PET/C textile fabrics treated with biosynthesized AgNPs

#### FTIR analysis

**Fig. 7:**
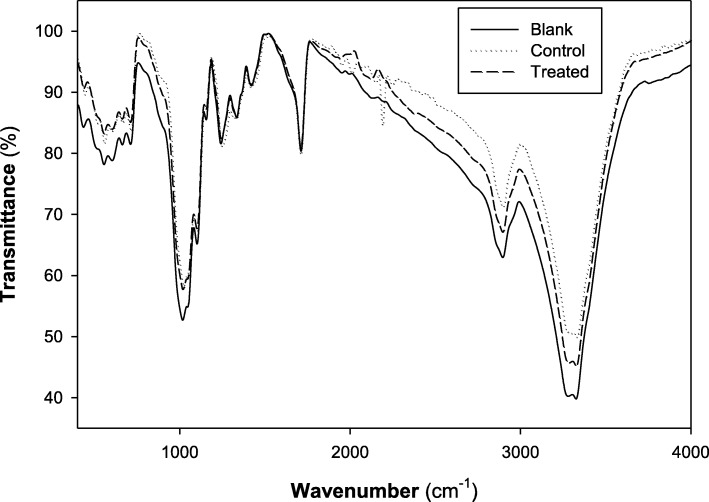
FTIR spectroscopy of the textiles treated with biosynthesized AgNPs. PET/C hydrolyzed by cellulase (Blank), PET/C hydrolyzed by cellulase and then treated by *A. fumigatus* CFF (Control), and PET/C hydrolyzed by cellulase, then treated by *A. fumigatus* biosynthesized AgNPs (Treated)

The FTIR spectrum (Fig. 7) of untreated PET/C blend fabric shows absorption bands at 1649–1712, 3408–3388, and 2317 cm^−1^ which are typical to those of ˃ C=O, O–H, and C–H stretching, respectively. New bands at 430, 440, and 460 cm^−1^ are observed in the spectrum of treated PET/C blend fabrics by AgNPs which can correspond to Ag–O bonds. During this study, we found that only activated surfaces were able to fix AgNPs from dispersion solutions.

#### SEM and EDAX analysis

**Fig. 8:**
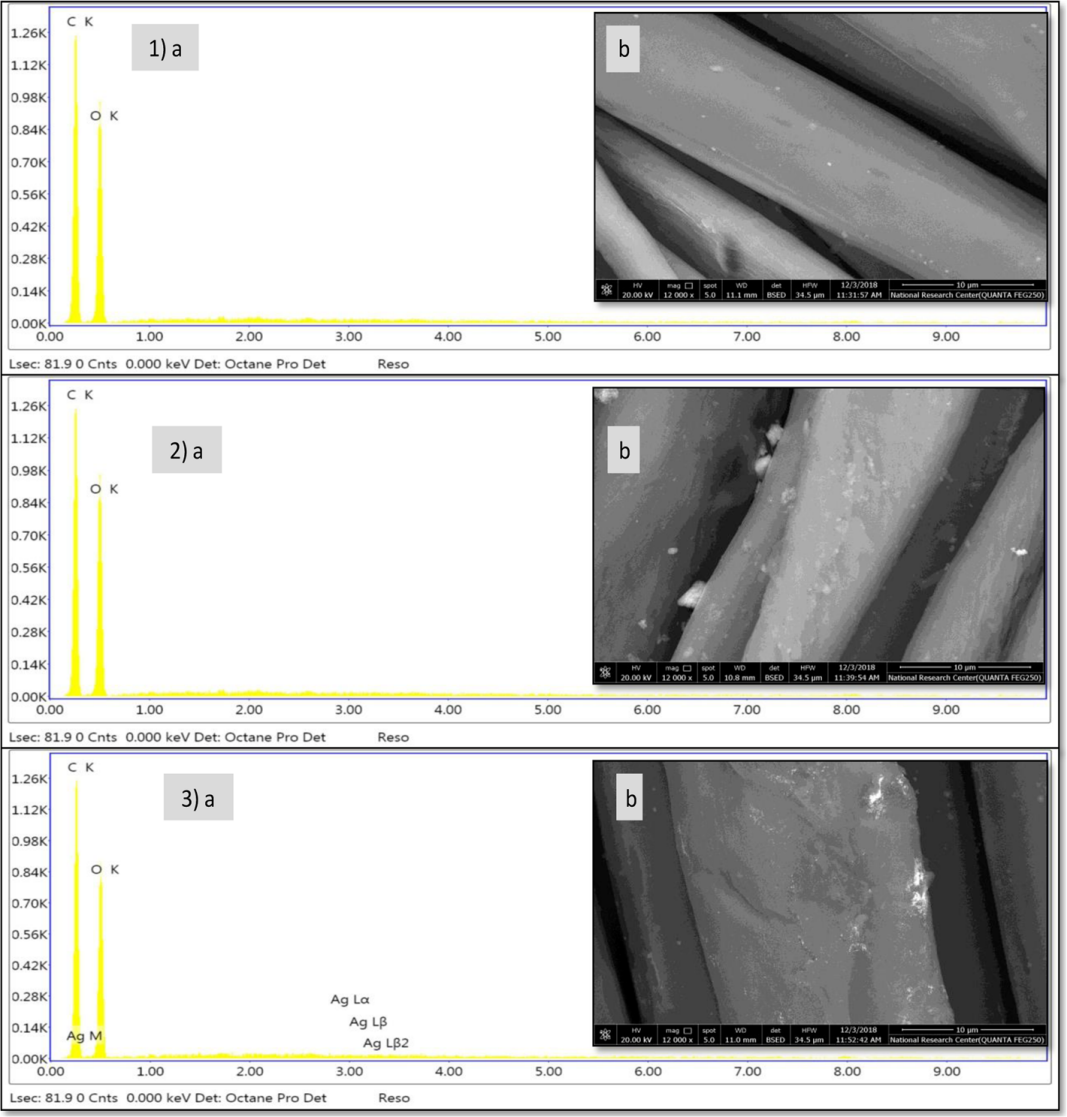
EDAX analysis (a) and SEM micrographs (b) of the PET/C textile treated with AgNPs biosynthesized by means of *A. fumigatus* CFF mediation. 1, Blank PET/C (not treated); 2, PET/C Control (treated with CFF); and 3, treated with biosynthesized nanosilver

The SEM and EDAX analysis micrographs of untreated and treated PET/C fabrics through immersion in *A. fumigatus* CFF and biosynthesized AgNPs solution are exposed in Fig. 8. As a quantitative and qualitative evaluation of elements found in untreated and treated fabrics, EDAX analysis was conducted (Fig. 8(a)). The resulted EDAX analysis established the incidence of elemental silver as weak energy peaks around 3.0 keV, verifying the adhesion of AgNPs successfully (Fig. 8(3b)), whereas there is no any detection of AgNPs in both untreated and control samples (Fig. 8(1a, 2a)). In addition, (EDAX) profile of the treated fabrics indicates the presence of silver by 0.25% (weight %) on the PET/C fabrics after five washing cycles. The SEM images in Fig. 8(b) display the differences in textures of fabrics before and after treatments, where the smooth structure appears in Fig. 8(1b), whereas the cotton fabrics after coating with *A. fumigatus* CFF show proteinaceous agglomerations on its surface Fig. 8(2b). After padding, the depositions of AgNPs on the fabrics are exposed in Fig. 8 (3b) as a thinner uniform surface layer.

#### Antimicrobial analysis

It is evident from EDAX profile of the treated PET/C fabrics by AgNPs that the presence of silver is 0.25% (weight %) on the PET/C fabrics. Even with this very low concentration, the treated fabrics exerted an antimicrobial activity against the tested microorganisms to give inhibition zone diameter of 15, 15, and 16 mm for *E. coli*, *B. mycoides*, and *C. albicans*, respectively.

## Discussion

Typically, AgNPs demonstrate distinctive optical properties due to their distinguishing optical resonance identified as surface plasmon resonance (SPR), which happens as an outcome of nanoparticle size and shape [4]. The UV–visible spectroscopy results using *A. fumigatus* DSM819 filtrate indicate the ability of *A. fumigatus* DSM819 to form AgNPs extracellulary through reduction of Ag^+^ to form the AgNPs. The distinct and typical SPR band for AgNPs was obtained in the region of 410 nm [23]. The ability of *A. fumigatus* DSM819 to form AgNPs is directly proportional to CFF ratio in the reaction mixture, where Othman et al. [23] referred that due to the increase of biomolecules participating in the metal reduction process to result in nanoparticles biosynthesis. It is worthy to mention that the capability of the produced enzyme and protein as reducing and stabilizing agents appeared in alkaline medium, and when the reaction was performed at pH values lower than 9.0, no SPR peak was obtained. Usually, the time necessary for complete metal ion reduction through the fungal biosynthesis of metal nanoparticles can vary from 24 to 124 h [11]. Here, in the current case, 90 min is the optimum time for AgNP formation which is due to the exceptional reducing potential of the *A. fumigatus* DSM819 CFF active components. The metal salt concentration has been revealed to influence nanoparticle biosynthesis effectively [28]. In the current case, the intensity of reaction color changed to reddish brown even at the lowest used concentration (0.5 mM) and become more dark reddish brown at higher concentrations of silver nitrate (1.0–1.5 mM). El-Rafie et al. [6] stated that by increasing silver nitrate concentration to high extent, the proteins and especially enzymes that present in the reaction mixture could be not sufficient for performing silver ion reduction to AgNPs and to stabilize them.

The highest counts of metallic silver nanocrystals typically appear at 3 keV in EDAX analysis as a result of their surface plasmon resonance [5], which agrees with the resulted EDAX analysis that established the incidence of elemental silver as strong signal energy peaks around 3.0 keV, and verifying the AgNPs biosynthesis successfully. The obtained TEM profile shows some individual silver particles in addition to some aggregates with dissimilar size ranges in indirect contact to ensure the nanoparticle stabilization by a capping agent [5, 23], with 90% of distribution below than 84.4 nm as obtained from DLS graph that confirm its nanoscale size. The FTIR spectrum declares the appearance of sharp peak at 3431 cm^−1^ that is thought to be overlapping –NH stretching vibration which is a typical phenomenon for proteins [16]. In addition, O–H stretching in flavonoids, alcohols, and phenolic compounds (3781, 3694, and 3431 cm^−1^) could be detected [17], and C–H stretching vibrations of methylene, methyl, and methoxy groups could be observed at 2923 and 2856 cm^−1^. The N–C=O amide bond of proteins due to carbonyl stretch in proteins was recorded at 1626 cm^−1^, while absorption peaks at 1121 and 1024 cm^−1^ can be due to stretching vibration of C–N aromatic and aliphatic amines [23].

The biosynthesized AgNPs have several featured applications like biomedical applications and antimicrobial surfaces due to its unique features and biocompatibility. The biosynthesized AgNPs through the mediation by *A. fumigatus* DSM819 CFF exerted an antimicrobial activity against the studied representative pathogenic microorganisms (*E. coli*, *B. mycoides*, and *C. albicans*) which depends on the microbe type and its cellular constituents [30]. Diverse routes of action can be used to understand the antimicrobial activity of AgNPs toward microorganisms. One of these mechanisms is AgNPs could attach to the negative charge on microbial cell surface which alter the cell wall and cell membrane properties, hence affecting osmoregulation, permeability, respiration, and electron transport [15]. As another approach, AgNPs can interact with cell constituents including DNA and proteins after penetrating microbial cell wall [2]. In addition, silver ions released by silver nanoparticles reason a bigger biocidal consequence depending on their dose and size [13, 24].

The results from in vitro cytotoxicity of the biosynthesized AgNPs revealed that there was a straight dose–response association with tested cells at tested concentrations. The biosynthesized AgNPs were able to restrain the cell line growth at low concentrations, where at a concentration of 54 μg/ml, they were able to inhibit 84.6, 60.8, 68.9, and 69.5% of HCT116, A549, MCF7, and PC3, respectively. Anand et al. [1] reported that a concentration close to 500 mg/ml of AgNPs biosynthesized by marine sediment significantly inhibits the HEp2 cell line growth by more than 85%. In this regard, some in vitro studies exposed the translocation of AgNPs in tumor cell line with a LC50 of 300 mg/ml [32]. Jacob et al. [9] stated that AgNPs could stimulate reactive oxygen groups and cause destruction to cellular components leading to cell fatality.

Evidently, enzymatic hydrolysis of PET/C blend fabrics before treatment with AgNPs induced a significant change in the chemical composition of the PET/C blend fabric surfaces. New bands at 430, 440, and 460 cm^−1^ are observed in the FTIR spectrum of treated PET/C blend fabrics by AgNPs which can correspond to Ag–O bonds. The presence of these bands can support the ionic character of the new band formed due the addition of AgNPs to activated fabrics with cellulase. During this study, we found that only activated surfaces were able to fix AgNPs from dispersion solutions. The resulted EDAX analysis of PET/C blend fabrics treated with AgNPs established the incidence of elemental silver as weak energy peaks around 3.0 keV, verifying the adhesion of AgNPs successfully. In addition, (EDAX) profile of the treated fabrics indicates the presence of silver by 0.25% (weight %) on the PET/C fabrics after five washing cycles. The SEM images display the differences in textures of fabrics before and after treatments, where the smooth structure appears in cellulase-treated sample, whereas the cotton fabrics after coating with *A. fumigatus* CFF show proteinaceous agglomerations on its surface, and the depositions of AgNPs on the fabrics as a thinner uniform surface layer after padding could be detected. Even with the detected very low concentration of nanosilver, the treated fabrics exerted an antimicrobial activity against *E. coli*, *B. mycoides*, and *C. albicans*. In this regard, El-Rafie et al. [6] reported that a diluted solution from *Fusarium solani* biosynthesized AgNPs imparts antibacterial activity to the cotton fabrics against both Gram-negative (*Escherichia coli*) and Gram-positive (*Staphylococcus aureus*) bacteria due to AgNP deposition onto cellulose molecules of cotton fabric and then via physical and chemical bonding.

## Conclusions

An efficient eco-friendly and inexpensive process for green synthesis of silver nanoparticles (AgNPs) through the fungal mediation of *Aspergillus fumigatus* DSM819 was achieved. AgNP biosynthesis using *A. fumigatus* DSM819 cell-free filtrate (CFF) was studied throughout investigating the most factors affecting the synthesis process. Under the optimum conditions, the biosynthesized AgNPs were of spherical shape with 90% of distribution below than 84.4 nm. The biosynthesized AgNPs exerted both antimicrobial and antitumor activities against the studied representative pathogenic microorganisms (*E. coli*, *B. mycoides*, and *C. albicans*) and in vitro human tumor cell lines. The treated PET/C fabrics by the biosynthesized AgNPs also exerted an antimicrobial activity. This green biosynthesis method is a non-toxic alternative to the traditional chemical and physical methods, and would be appropriate for biological large-scale production and prospective treatments.

## Data Availability

The datasets used and/or analyzed during the current study are available from the corresponding author on reasonable request.

## Abbreviations

A549: Lung carcinoma cell line
AgNPs: Silver nanoparticles
BJ1: Normal skin fibroblast
CFF: Cell-free filtrate
DLS: Dynamic light scattering
DMSO: Dimethyl sulfoxide
EDAX: Energy dispersive X-ray
FTIR: Fourier transform infrared
HCT116: Colon cell line
IC_50_: Lethal concentration which causes the death of 50% of cells
IC_90_: Lethal concentration which causes the death of 90% of cells
MCF7: Caucasian breast adenocarcinoma
MTT: 3-(4,5-Dimethylthiazol-2-yl)-2,5-diphenyl tetrazolium bromide
PC3: Prostate cell line
PET/C: Polyester/cotton blend
SEM: Scanning electron microscope
SPR: Surface plasmon resonance
TEM: Transmitting electron microscopic
