# Correction to: Biosynthesis and characterization of silver nanoparticles induced by fungal proteins and its application in different biological activities

**DOI:** 10.1186/s43141-020-0022-3

**Published:** 2020-02-11

**Authors:** Abdelmageed M. Othman, Maysa A. Elsayed, Naser G. Al-Balakocy, Mohamed M. Hassan, Ali M. Elshafei

**Affiliations:** 1 Microbial Chemistry Department, Genetic Engineering and Biotechnology Research Division, National Research Centre, 33 El Bohouth St., Dokki, Giza, 12622 Egypt; 2 Protein and Manmade Fibers Department, Textile Research Division, National Research Centre, 33 El Bohouth St., Dokki, Giza, 12622 Egypt

**Correction to: J Genet Eng Biotechnol (2019) 17:8**

**https://doi.org/10.1186/s43141-019-0008-1**

In the publication of this article [1], the title of Figure 6 was missing. The original article has been corrected.

The error:

Fig. 6 (1) AgNPs sample formed at pH 6.0, (2) at pH 8.0, (3) at pH 10.0, (4) at pH 12.0, and (5) cell free filtrate

It should instead read:

Fig. 6 Antimicrobial activity of the AgNPs biosynthesized by means of *A. fumigatus* CFF mediation. (1) AgNPs sample formed at pH 6.0, (2) at pH 8.0, (3) at pH 10.0, (4) at pH 12.0, and (5) cell free filtrate
